# R-Fluoxetine Increases Melanin Synthesis Through a 5-HT1A/2A Receptor and p38 MAPK Signaling Pathways

**DOI:** 10.3390/ijms20010080

**Published:** 2018-12-25

**Authors:** Li Liu, Mengsi Fu, Siran Pei, Liangliang Zhou, Jing Shang

**Affiliations:** 1 State Key Laboratory of Natural Medicines, China Pharmaceutical University, Nanjing 210009, China; lilcpu@hotmail.com (L.L.); 15151826073@163.com (M.F.); peisiran@hotmail.com (S.P.); zhoulliang@163.com (L.Z.); 2 Jiangsu Key Laboratory of TCM Evaluation and Translational Research, China Pharmaceutical University, Nanjing 210009, China

**Keywords:** r-fluoxetine, zebrafish, melanin

## Abstract

Fluoxetine, a member of the class of selective serotonin reuptake inhibitors, is a racemic mixture and has an anxiolytic effect in rodents. Previously, we have shown that fluoxetine can up-regulate melanin synthesis in B16F10 melanoma cells and normal human melanocytes (NMHM). However, the role of r-fluoxetine and s-fluoxetine, in the regulation of melanin synthesis, is still unknown. Here, we show how r-fluoxetine plays a critical role in fluoxetine enhancing melanogenesis, both in vivo and vitro, by up-regulating tyrosinase (TYR) and the microphthalmia-associated transcription factor (MITF) expression, whereas, s-fluoxetine does not show any effect in the vivo and vitro systems. In addition, we found that r-fluoxetine induced melanin synthesis through the serotonin1A receptor (5-HT1A) and serotonin 2A receptor (5-HT2A). Furthermore, r-fluoxetine increased the phosphorylation of p38 mitogen-activated protein kinase (p38 MAPK), without affecting the phosphorylation of extracellularly responsive kinase (ERK1/2) and c-Jun N-terminal kinase (JNK). These data suggest that r-fluoxetine may be used as a drug for skin hypopigmentation disorders.

## 1. Introduction

Melanin is produced in melanocytes in the basal layer of the epidermis and provides protection to the human skin, from ultraviolet radiation [1,2,3]. It is synthesized through a complex biochemical process and involves more than hundred distinct genes [4]. There are three required enzymes for melanin synthesis, including tyrosinase (TYR), tyrosinase-related protein 1 (TRP-1), and dopachrome tautomerase (DCT, TRP-2) [5]. These three enzymes are transcriptionally regulated by the microphthalmia-associated transcription factor (MITF), the master gene of melanocyte differentiation [6,7]. Skin cells are capable of producing neuropeptides, neurotransmitters, hormones, and corresponding functional receptors [8]. It has been reported that l-tyrosine and l-dopa play a role in the regulation of melanogenesis, and function as hormone-like regulators [9].

Zebrafish has emerged as a highly advantageous vertebrate animal system, for screening melanogenic inhibitors and stimulators, because of its small size, transparent embryos, and physiological similarity to mammals [10,11]. Furthermore, one advantage of the zebrafish is that it simultaneously measures compound toxicity [12]. We can determine the toxicity of compounds through the mortality, morphological malformation, and cardiac function.

Serotonin (5-hydroxytryptamine, 5-HT), a neurotransmitter whose actions are mediated through corresponding receptors, includes seven families (5-HT_1–7_ receptors), with at least twenty-one subtypes. Skin cells not only express serotonin receptors including 5-HT_1A_, 5-HT_1B_, 5-HT_2A_, 5-HT_2B_, 5-HT_2C_, and 5-HT_7_ receptors, but can also produce and metabolize serotonin [13,14,15]. Fluoxetine, a member of the class of selective serotonin reuptake inhibitors, is a racemic mixture and has an anxiolytic effect in rodents [16,17]. In our previous studies, we have found that fluoxetine can up-regulate melanin synthesis in B16F10 melanoma cells and normal human melanocytes [18]. However, the role of the r/s-fluoxetine, in the regulation of melanin synthesis, is still unknown. In this study, we investigated the effect of r-fluoxetine and s-fluoxetine on melanogenesis, using the B16F10 melanoma cells and zebrafish models.

## 2. Results

### 2.1. Effect of R/S-Fluoxetine on Tyrosinase Activity and Melanin Synthesis in B16F10 Cells

**Figure 1:**
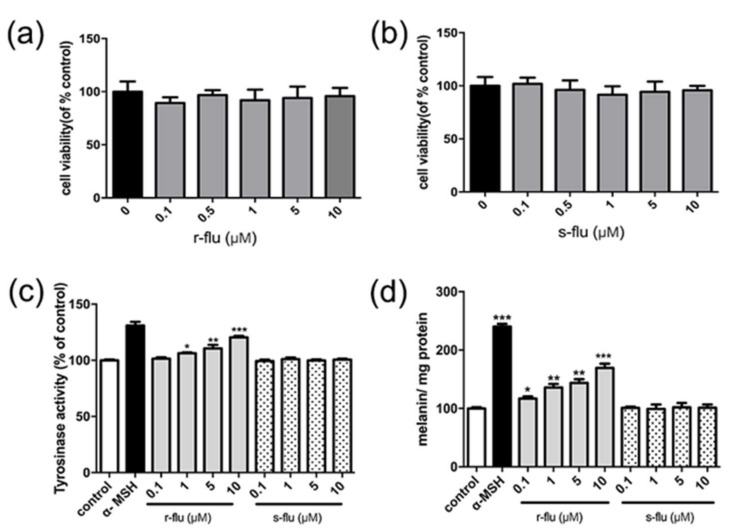
Effect of the r-fluoxetine and s-fluoxetine on the B16F10 cells viability, tyrosinase activity, and melanin content. B16F10 cells were incubated with r-fluoxetine and s-fluoxetine, at various concentrations, for 48 h, and the cell viability was examined by an MTT assay, r-fluoxetine (**a**) and s-fluoxetine (**b**). Tyrosinase activity (**c**) and melanin contents (**d**) were performed, as described in the Materials and Methods section. * *p* < 0.05, ** *p* < 0.01, *** *p* < 0.001, compared with control.

To determine the effect of r-fluoxetine and s-fluoxetine on melanogenesis, a 3-(4, 5-dimethylthiazol)-2, 5-diphenyl tetrazolium bromide (MTT) assay was done first. As shown in Figure 1a,b, r/s-fluoxetine were not cytotoxic to the B16F10, cells at concentrations of 0.1–10 μM. Thus, to investigate the effect of r/s-fluoxetine on tyrosinase and melanin synthesis, B16F10 cells were then exposed to r/s-fluoxetine, from 0.1–10 μM. As shown in Figure 1c,d, r-fluoxetine significantly upgraded the tyrosinase activity and melanin contents in a concentration-dependent manner, in the B16F10 cells. However, as compared to the control group (medium only), treatment with s-fluoxetine had no effect on the melanin contents in the B16F10 cells.

### 2.2. Effect of R/S-Fluoxetine on MITF and Tyrosinase Protein Expression in B16F10 Cells

**Figure 2:**
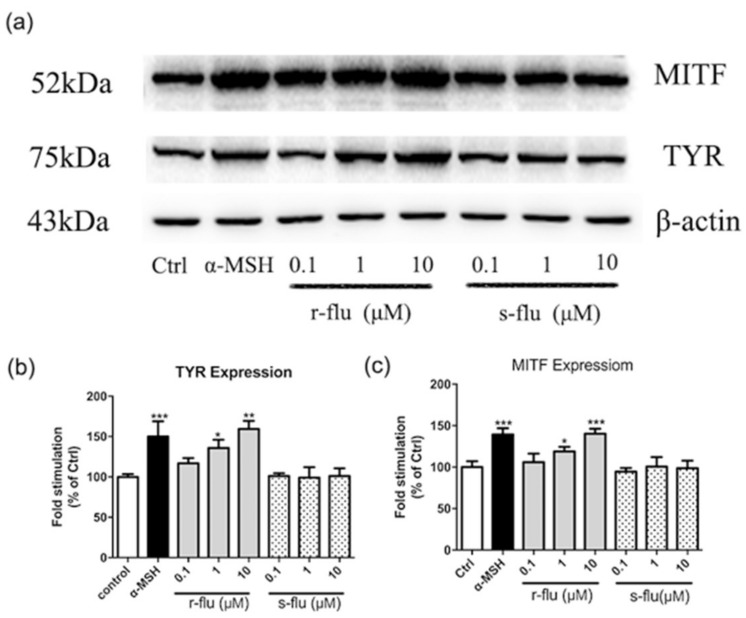
Effect of r-fluoxetine and s-fluoxetine on the expression of the tyrosinase (TYR) and the microphthalmia-associated transcription factor (MITF) in B16F10 cells. (**a**) Western Blot assays were performed to examine MITF and TYR expression levels. (**b**,**c**) Densitometry scanning of the band densities were utilized to measure the expression of proteins by the Quantity One software. Bars indicate the means ± SEM of three independent experiments. * *p* < 0.05, ** *p* < 0.01, *** *p* < 0.001, compared vs. control.

**Figure 3:**
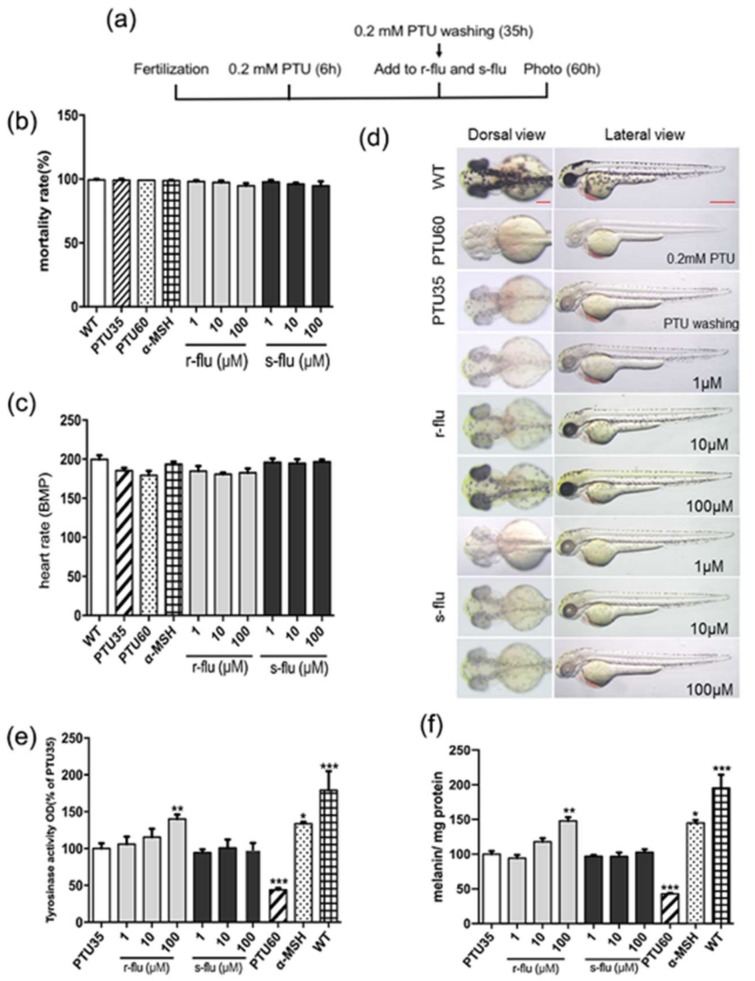
Effect of r/s-fluoxetine on tyrosinase activity and melanin synthesis in zebrafish. (**a**) Schematic representation for the schedule of pigmentation rescue study. (**b**) Mortality rate was calculated by calculating the normally developed embryos at various concentrations for 48 h. (**c**) The heart-beating rate was measured at 60 hpf (hours post-fertilization), under the stereomicroscope. (**d**) Synchronized embryos were treated with 0.2 mM 1-phenyl-2-thiourea (PTU) at 6 hpf. r-fluoxetine and s-fluoxetine was added and incubated for a further 25 h, after a PTU wash, at 35 hpf. Scale bar, 200 μm. (**e**) Tyrosinase activity and (**f**) melanin contents of about thirty synchronized embryos, collected and dissolved in cold lysis buffer. After centrifugation, 10 μg of the total protein was incubated with 0.1% of l-dopa, as described in Section 4. All experiments were repeated three times. Data were analyzed by one-way analysis of variance (ANOVA), followed by a post hoc Tukey test. Bars indicate the means ± SEM of the three independent experiments. * *p* < 0.05, ** *p* < 0.01, *** *p* < 0.001, compared with the PTU35 (control).

Since r-fluoxetine increases the tyrosinase activity and melanin synthesis, we further investigated whether r-fluoxetine affected the expression of the TYR and the MITF, which plays a key role in the tyrosinase expression. As shown in Figure 2, B16F10 cells exposed to various concentrations of r-fluoxetine, had significant increases in the TYR expression. Thus, the effect of r-fluoxetine on MITF expression, was investigated. As shown in Figure 3, the expression of MITF was found to be up-regulated, after treatment with various concentrations of r-fluoxetine. However, there were no obvious effects of the s-fluoxetine on the TYR and the MITF expression.

### 2.3. Effect of R/S-Fluoxetine on Tyrosinase Activity and Melanin Synthesis in Zebrafish

The early zebrafish embryo rapidly absorbs small molecule compounds into the skin, which is transparent, allowing for a simple observation of the pigmentation process, without complicated experiments. To determine the effect of r-fluoxetine and s-fluoxetine on melanogenesis, we examined zebrafish mortality in the r/s-fluoxetine condition treatments. As shown in Figure 3b,c, r/s-fluoxetine had no obvious cytotoxic effects on the zebrafish, at concentrations of 1–100 μM. Thus, as shown in Figure 3d, r-fluoxetine marked the stimulation of the zebrafish body pigmentation. To determine the molecular targets of the r-fluoxetine, we measured the total melanin content and tyrosinase activity, using whole extracts of zebrafish. As shown in Figure 3e,f, the r-fluoxetine significantly upgraded the tyrosinase activity and melanin contents, in a concentration-dependent manner, in the zebrafish.

### 2.4. Effect of R/S-Fluoxetine on the Promoter Activities of Mitfa and Tyrp1a in the Zebrafish

**Figure 4:**
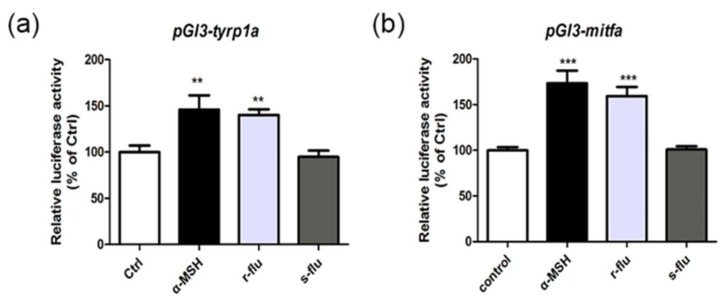
B16F10 cells were transfected with luciferase reporter constructs and treated with the r/s-fluoxetine 10 μM for 24 h. (**a**) pGL3-tyrp1a and (**b**) pGL3-mitfa. Results shown are means ± SEM and representative of three independent experiments. Data were analyzed by ANOVA, followed by a post hoc Tukey test. Bars indicate the means ± SEM of the three independent experiments. ** *p* < 0.01, *** *p* < 0.001, compared with the control group.

To further investigate the effect of the r/s-fluoxetine on the activities of the mitfa and the tyrp1a promoters, we used a pGL3-sv40 luciferase reporter plasmid, comprising the zebrafish mitfa and tyrp1a proximal promoter to perform luciferase assays in the B16F10 cells. As shown in Figure 4a,b, the r-fluoxetine significantly increased in the activity of the mitfa:luciferase and the tyrp1a:luciferase, as compared to the control:luciferase.

### 2.5. Effect of R/S-Fluoxetine on the GFP (Green Fluorescent Protein) Expression in the Tyrp1a:eGFP and Mitfa:eGFP Zebrafish

**Figure 5:**
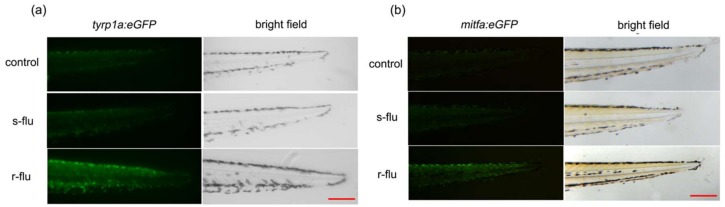
Effect of the r-fluoxetine and the s-fluoxetine on the gfp expression of the tyrp1a:eGFP and the mitfa:eGFP zebrafish. (**a**) tyrp1a:eGFP zebrafish and (**b**) mitfa:eGFP zebrafish Synchronized embryos were treated with 0.2 mM 1-phenyl-2-thiourea (PTU) at 6 hpf. r-fluoxetine (100 μM) and s-fluoxetine (100 μM) were added and incubated for a further 25 h, after a PTU wash at 35 hpf. Scale bar, 100 μm.

In the tyrp1a:eGFP zebrafish line, the GFP expression, perfectly matched the tyrp1a expression. As shown in Figure 5a,b, the expression of the green fluorescent protein of the tyrp1a:GFP and the mitfa:eGFP, were significantly up-regulated by the r-fluoxetine, whereas, no effect was observed for the s-fluoxetine on the tyrp1a:eGFP zebrafish. 

### 2.6. R-Fluoxetione Induce Melanin Synthesis through 5-HT1A Receptor and 5-HT2A Receptor

**Figure 6:**
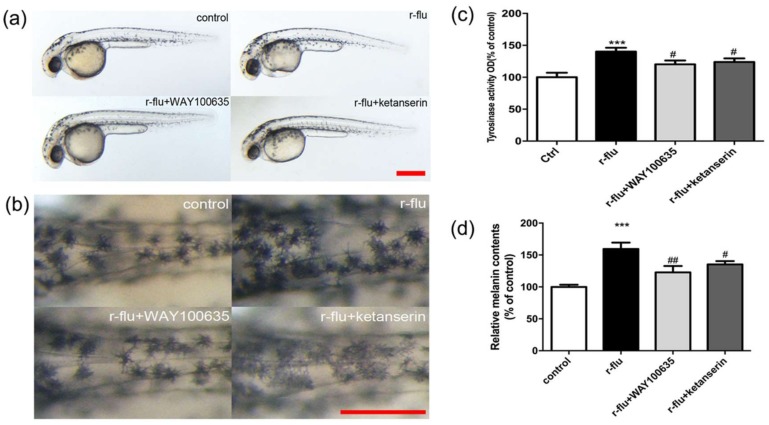
Effect of the WAY100635 and the ketanserin on the melanin contents in the r-fluoxetine-induced zebrafish pigmentation. Synchronized embryos were treated with r-flu (100 μm), WAY100635 (10 μM), and ketanserin (10 μM), at 6 hpf and incubated for further 30 hpf. The effect on the pigmentation of the zebrafish were photographed under the stereomicroscope. (**a**) Lateral view of embryos at 36 hpf, (**b**) dorsal view of embryos at 36 hpf. Scale bar, 200 μm. (**c**) Tyrosinase activity and (**d**) melanin contents were performed, as described in the Materials and Methods section. Data were analyzed by a one-way analysis of variance (ANOVA) followed by post hoc Tukey test. Bars indicate the means ± SEM of the three independent experiments. *** *p* < 0.001, compared with the control; # *p* < 0.05, ## *p* < 0.01, compared with r-flu group.

It has been reported that fluoxetine promotes melanogenesis in B16F10 cells, by directly activating the 5-HT1A receptor rather than by serotonin reuptake, and that the 5-HT2A receptor is involved in the melanogenesis. Thus, we investigated which r-fluoxetine promotes melanin synthesis through the HTR (serotonin receptor). As shown in Figure 6a–d, WAY100635 and ketanserin were able to reverse the increase in melanin synthesis induced by the r-fluoxetine in the B16F10 cells and the zebrafish. Thus, the r-fluoxetine possibly induces a melanin synthesis through the 5-HT1A receptor and the 5-HT2A receptor.

### 2.7. Effect of the R-Fluoxetine on Phosphorylation of the p38 MAPK, ERK1/2, and JNK in the B16F10 Cells

**Figure 7:**
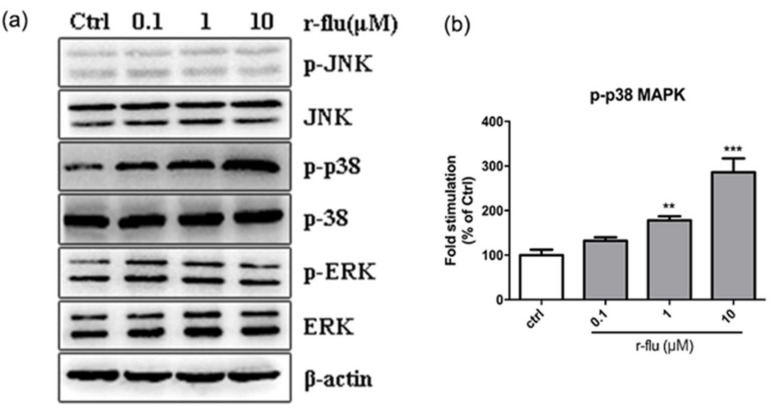
Effect of the r-fluoxetine on the expression of the MAPK signaling pathways in the B16F10 cells. (**a**) Western Blot assays were performed to examine the p38 MAPK, ERK, and JNK expression levels. (**b**) Densitometry scanning of the band densities of the p38 MAPK were utilized to measure the expression of proteins by Quantity One software. Bars indicate the means ± SEM of the three independent experiments. ** *p* < 0.01, *** *p* < 0.001 vs. control group.

To further elucidate the mechanism underlying the effect of the r-fluoxetine on melanogenesis, the mitogen-activated protein kinase (MAPK) intracellular signal transduction cascade was detected by Western blotting. As shown in Figure 7, the phosphorylation of the p38 MAPK was increased after adding r-fluoxetine, whereas, no effect was found in the phosphorylation of the extracellularly responsive kinase (ERK1/2), c-Jun N-terminal kinase (JNK), and the total protein levels of the p38. r-fluoxetine increased the phosphorylation of the p38 MAPK in a concentration-dependent manner.

## 3. Discussion

Fluoxetine is a selective serotonin reuptake inhibitor used to treat depression disorders [19]. It has been reported that antidepressant drug reactions result in the hyperpigmentation of the skin and fluoxetine can promote hair follicle pigmentation [20]. In addition, there are side effects when fluoxetine is used as a drug for skin hypopigmentation disorders. However, these can be effectively reduced by skin administration of fluoxetine. Racemic fluoxetine consists of two stereoisomers. r-fluoxetine is mainly used as an antidepressant, while s-fluoxetine is used to treat migraine prophylaxis [21,22]. It has been recognized that component stereoisomers of medications can have markedly different pharmacodynamics effect profiles from the racemate. Usually only a single enantiomer was effective against the disease [23,24]. According to our group’s previous studies, fluoxetine can up-regulate melanin synthesis in B16F10 melanoma cells and in normal human melanocytes. Thus, it is suggested that the influence of the r/s-fluoxetine, on the melanogenic activity, may be different. As shown in Figure 1, when treated with r-fluoxetine, tyrosinase activity and melanin were significantly upgraded, while there were no obvious effects after being treated with s-fluoxetine.

We further investigated the effect of the r/s-fluoxetine on the expression of TYR and MITF, which play an important role in melanogenesis. TYR plays an important role in melanogenesis, by catalyzing the hydroxylation of the tyrosine to l-dopa, and the oxidation of the l-dopa to the dopa quinone [25]. MITF, as the major transcriptional regulator of tyrosinase, plays an important role in melanogenesis [26]. As shown in Figure 2, r-fluoxetine significantly increased the MITF and tyrosinase levels, at 48 h, in the B16F10 cells.

As the results in vitro may be different from that in vivo, we brought wild-type zebrafish and transgenic zebrafish, as the in vivo model, to evaluate the activity of the melanogenesis of the r/s-fluoxetine. The 1-phenyl-2-thiourea (PTU) is a tyrosinase inhibitor used to inhibit the production in zebrafish [27]. Our results showed that the exposure of the larvae to the r-fluoxetine, increased the pigmentation production in a concentration-dependent manner, without toxicity in the exposed zebrafish. It has been reported that we can analyze the expression of the target gene by detecting the green fluorescent protein of the transgenic zebrafish [28]. Mitfa are the melanocytic markers and the tyrp1a expression perfectly matched the melanocyte development [29]. We chose mitfa and tyrp1a as promoters, for further study. To determine the tyrp1a and mitfa expression, we used Tol2 transposon cassettes containing these non-coding sequence elements, linked to the GFP. Hence, we generated two GFP reporter transgenic lines, using 2001 bps of the tyrp1a promoter and 835bps of the mitfa promoter [30,31]. We, therefore, examined the influence of the r/s-fluoxetine treatment on tyrp1a:eGFP and the mitfa:eGFP zebrafish. As our data show, after treatment with the r-fluoxetine at 100 μM, the gfp expression was significantly up-regulated, while, this was not changed, after treatment with the s-fluoxetine. Additionally, the r-fluoxetine significantly increased the promoter activities of both the mitfa and the tyrp1a, suggesting that the r-fluoxetine affected both the expression and activity of the mitfa and the tyrp1a, in the melanogenesis. Moreover, results of the promoter activity assay (Figure 5a,b) of the mitfa and the tyrp1a also supported that the r-fluoxetine could directly regulate the mitfa and the tyrp1a activity.

Previous data showed that fluoxetine promotes melanogenesis involved in serotonin 1A receptor(5-HT1A). According to reports, fluoxetine inhibits serotonin reuptake and serotonin 2A receptor(5-HT2A) are involved in serotonin or 5-HT2A agonist-induced melanogenesis [32,33]. Therefore, we used a selective 5-HT1A receptor antagonist (WAY100635) and a selective 5-HT2A receptor antagonist (ketanserin), to evaluate the involvement of the 5-HT1A receptor and 5-HT2A receptor in the r-fluoxetine up-regulated melanogenesis. As shown in Figure 6, both WAY100635 and ketanserin were able to reverse the increase in melanin content induced by the r-fluoxetine. Thus, this finding indicated that the r-fluoxetine promotes melanogenesis, by activating the 5-HT1A receptor and 5-HT2A receptor.

It has been reported that the MAP kinase family, including the ERK, JNK, and p38 MAPKs, plays an important role in the regulation of melanogenesis [34,35]. Activations of the ERK and the JNK/SAPK pathways were related to the down-regulation of melanogenesis. Phosphorylation of the p38 can activate MITF, which, in turn, can induce the melanocyte differentiation and melanogenesis. Thus, we investigated the influence of the r-fluoxetine treatment on the activation of the p38, JNK, and the ERK MAPKs, to further understand the molecular mechanisms involved in the pigmentation of the r-fluoxetine. As shown in Figure 7, r-fluoxetine (0.1–10μM) increased the p38 phosphorylation, without affecting the phosphorylation of the ERK and the JNK. The p38 MAPK cascade can activate the MITF expression, and the MITF transcriptionally regulates the TYR, TRP-1, and the TRP-2. Our data showed that activating the p38 phosphorylation and then inducing the expression of the MITF and the TYR, TRP-1, and the TRP-2 contributed to the melanogenic effect of the r-fluoxetine. These results showed the molecular mechanisms underlying the melanogenic activity of the r-fluoxetine.

**Figure 8:**
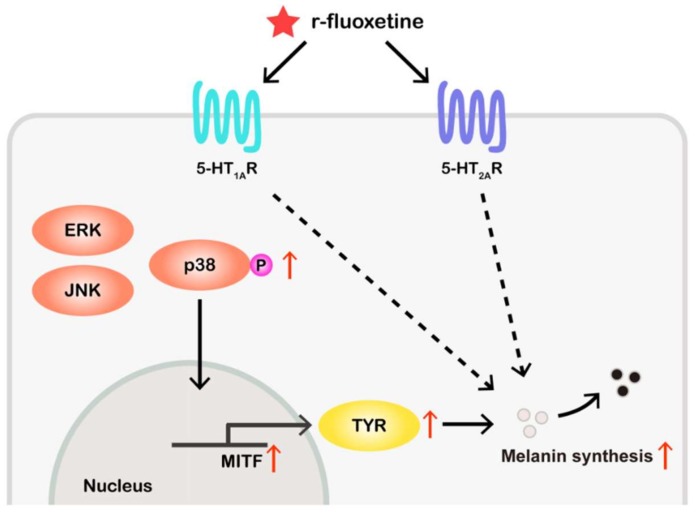
Schematic description of the changes in melanin synthesis induced by r-fluoxetine. Red arrow define the activity of r-fluoxetine, black arrow define the direct stimulatory modification, dotted arrow define the tentative stimulatory modification.

In summary, we showed that r-fluoxetine plays a critical role in fluoxetine-enhancing melanogenesis, both in vivo and vitro, by up-regulating the tyrosinase and the MITF expression. However, the s-fluoxetine did not show any effects in vivo and vitro. In addition, we found that the r-fluoxetine induced melanin synthesis through the serotonin1A receptor and the serotonin 2A receptor (Figure 8). Furthermore, the r-fluoxetine increased the phosphorylation of the p38 MAPK, without affecting the phosphorylation of the ERK1/2 and the JNK. This data suggests that r-fluoxetine could be used as a drug for skin hypopigmentation disorders. In addition, our data indicates that the tyrp1a:eGFP transgenic zebrafish could be a new model for screening and evaluating melanogenic regulatory compounds.

## 4. Materials and Methods

### 4.1. Cell Culture and Materials

The melanoma cell line B16F10 was obtained from the Cell Bank of the Chinese Academy of Sciences, Shanghai, China and grown in Dulbecco’s Modified Eagle’s Medium (DMEM, Gibco, CA, USA), supplemented with 10% (*v*/*v*) heat-inactivated fetal bovine serum (FBS, Gibco, CA, USA), 100 U/mL penicillin, 100 μg/mL streptomycin (Gibco, CA, USA), at 37 °C, in a humidified 5% CO_2_ incubator [36].

### 4.2. Cell Viability Assay

The viability of the cultured cells was determined by 3-(4, 5-dimethylthiazol)-2, 5-diphenyl tetrazolium bromide (MTT) assay. In brief, after different concentrations of the r/s-fluoxetine were incubated for 48 h, 10 μL of 5 mg/mL MTT in PBS, was added into each well, and incubated at 37 °C, for 4 h. Following the MTT removal, 100 μL of the DMSO (dimethyl sulfoxide) was added to each well. The absorbance was measured at 570 nm with a spectrophotometer.

### 4.3. Melanin Measurement and Tyrosinase Assay

Tyrosinase activity, as the dopa oxidase, was measured by the rate of l-dooa oxidation as has been previously reported [37]. B16F10 cells were treated with r-fluoxetine and s-fluoxetine, for 48 h, then washed with PBS, three times, and harvested by centrifugation, at 12,000 rpm, for 15 min. Then, 100 μL NaOH containing 10% DMSO was added to dissolve the melanin for 2 h, in 80 °C. The absorbance of melanin at 405 nm was measured. To estimate the effect of the r-fluoxetine on the tyrosinase activity, the B16F10 cells were lysated, with the cell lysis buffer, and then mixed with 100 μL 0.1% l-dopa in 0.1 M PBS (pH 6.8). The absorbance was measured at 475 nm, using a microplate reader.

### 4.4. Western Blot Analysis

B16F10 cells were washed with PBS and then dissolved in 100 μL lysis buffer for 20 min. After centrifugation at 12,000 rpm for 1 min, the protein suspension was obtained by collecting the upper solution. Then, 30 μg proteins were separated by a 10% SDS-PAGE gel and transferred to the polyvinylidende fluoride (PVDF) membranes. The membranes were blocked with 2% albumin from bovine serum (BSA) for 1 h, then washed with tris-buffered saline (TBS) containing 0.1% Tween20 (TBST) three times, and incubated with primary antibodies targeting MITF (BS1550), TYR (C-19) (SC7833), p-p38 (Thr180/Tyr182) (CST4631), p38 (CST9212), p-ERK1/2 (Thr202/Tyr204) (CST4376), ERK1/2 (CST4695), p-JNK (Thr183/ Tyr185) (CST9251), JNK (CST9258), p-CREB (Ser133) (CST9198), CREB (CST9197), and b-actin (CST3700) with TBST. After the reaction with the second antibody, an enhanced chemiluminescence detection system was used to visualize the proteins. Densitometric analysis of the bands were performed using the Quantity One (Bio-Rad, Hercules, CA, USA). Western blot assay results represented at least three independent experiments.

### 4.5. Zebrafish Maintenance

The experiments involved in the zebrafish were approved by the Animal Experimentation Ethics Committee at the China Pharmaceutical University. Wild-type AB line zebrafish were obtained from the Model Animal Research Center (MARC, Nanjing, China) and maintained in a circulating aquaculture system, according to the standards described in The Zebrafish Book. Embryos were incubated at 28.5 °C and staged, according to the description by Kimmel et al. [38].

### 4.6. Measurement of the Pigmenting Activity in the Zebrafish

Synchronized embryos were collected, thirty embryos per well, in 6-well plates, containing 5 mL embryo medium. r/s-fluoxetine were dissolved in 0.1% DMSO. In experiments, 0.2 mM PTU was administered to the zebrafish, from 6 to 35 hpf, then washed and bathed immediately, in the medium, at 35 hpf, in the presence of the r/s-fluoxetine, at the indicated concentrations. The effects on the pigmentation of the zebrafish were observed under the stereomicroscope.

### 4.7. The Luciferase Assay

The promoter of mitfa, which includes a BglII restriction site and the KpnI restriction, was amplified using the primers: F: 5′-CATCGGGGTACCCTCGAGAACTTCCCTGCTATTTAT-3′ and R: 5′-GGCCGAAGATCTGTTCAACTATGTGTTAGCTTC-3′. The promoter of tyrp1a, which includes a KpnI restriction site and the XhoI restriction, was amplified using the primers: F: 5′-CATCGGGGTACCGAGAGGATCAGGAGAAGAAAA-3′ and R: 5′-CCGCTCGAGCGCAGGAAATCTAGAAATAAG-3′. The PCR product were inserted into the pGL3-basic vector (Promega, Madison, WI, USA) called “pGl3-mitfa” and “pGL3-tyrp1a”. The construct was confirmed by sequencing. B16F10 cells was seeded into each well of a 24-well plate, 24 h prior to transfection. Transfection mixtures contained 2 μL Lipofectamine 2000 reagent (Invitrogen, Carlsbad, CA, USA), 60 ng pGl3-mitfa plasmid, and 1.2 ng renilla (an internal control, Promega, Madison, WI, USA), per well, in a 24-well plate and the mixtures were transfected into the B16F10 cells. Renilla and the firefly luciferase activities were measured using the Dual-Glo Luciferase Reporter Assay System (Promega, Madison, WI, USA), according to the manufacturer’s protocols.

### 4.8. Measurement of the Heart-Beating Rate and the Mortality Rate

The heart-beating rate of zebrafish was measured at 60 hpf, to determine the compound toxicity [39]. The embryos were embedded in 3% methylcellulose and the number of sequential atrial and ventricular contractions in 20 s intervals were counted. Mortality rate was measured at 60 hpf, under a dissecting microscope.

### 4.9. Statistical Analysis

Data were expressed as means ± SEM. The statistical analyses of the results were performed with one-way ANOVA and Tukey’s test, for comparing all pairs of columns, *p* < 0.05, 0.01 and 0.001 were accepted as statistically significant.

## 5. Conclusions

Our result showed that the r-fluoxetine played a critical role in the fluoxetine enhancing melanogenesis, both in vivo and vitro, by up-regulating the tyrosinase and MITF expression. However, s-fluoxetine did not show any effect in the vivo and vitro system. These data suggest that r-fluoxetine may be used as a drug for skin hypopigmentation disorders. In addition, our presented data indicated that the tyrp1a:eGFP transgenic zebrafish might be a new model for screening and evaluating melanogenic regulatory compounds.

## Abbreviations

TYR	tyrosinase
MITF	microphthalmia-associated transcription factor
5-HT1AR	serotonin1A receptor
5-HT2AR	serotonin 2A receptor
PTU	1-phenyl-2-thiourea
PTU35	1-phenyl-2-thiourea 35 hpf
PTU60	1-phenyl-2-thiourea 60 hpf
MAPK	mitogen-activated protein kinase
TBS	tris- buffered saline
V/V	volume/volume
DMSO	dimethyl sulfoxide
hpf	hours post-fertilization
MTT	3-(4, 5-dimethylthiazol)-2, 5-diphenyl tetrazolium bromide
